# The Effect of Water Deficit on Two Greek *Vitis vinifera* L. Cultivars: Physiology, Grape Composition and Gene Expression during Berry Development

**DOI:** 10.3390/plants10091947

**Published:** 2021-09-18

**Authors:** Anastasios Alatzas, Serafeim Theocharis, Dimitrios-Evangelos Miliordos, Konstantina Leontaridou, Angelos K. Kanellis, Yorgos Kotseridis, Polydefkis Hatzopoulos, Stefanos Koundouras

**Affiliations:** 1 Laboratory of Molecular Biology, Department of Biotechnology, Agricultural University of Athens, 75 Iera Odos, 11855 Athens, Greece; aalatzas@aua.gr; 2 Laboratory of Viticulture, School of Agriculture, Aristotle University of Thessaloniki, 54124 Thessaloniki, Greece; sertheo@agro.auth.gr; 3 Laboratory of Enology and Alcoholic Drinks, Department of Food Science and Human Nutrition, Agricultural University of Athens, 75 Iera Odos, 11855 Athens, Greece; dim.miliordos@gmail.com (D.-E.M.); ykotseridis@aua.gr (Y.K.); 4 Laboratory of Pharmacognosy, Department of Pharmaceutical Sciences, Aristotle University of Thessaloniki, 54124 Thessaloniki, Greece; kleontar@pharm.auth.gr (K.L.); kanellis@pharm.auth.gr (A.K.K.)

**Keywords:** grapes, Agiorgitiko, Assyrtiko, drought stress, anthocyanins, phenols, phenylpropanoid and terpenoid pathway

## Abstract

Plants are exposed to numerous abiotic stresses. Drought is probably the most important of them and determines crop distribution around the world. Grapevine is considered to be a drought-resilient species, traditionally covering semiarid areas. Moreover, in the case of grapevine, moderate water deficit is known to improve the quality traits of grape berries and subsequently wine composition. However, against the backdrop of climate change, vines are expected to experience sustained water deficits which could be detrimental to both grape quality and yield. The influence of water deficit on two Greek *Vitis vinifera* L. cultivars, ‘Agiorgitiko’ and ‘Assyrtiko’, was investigated during the 2019 and 2020 vintages. Vine physiology measurements in irrigated and non-irrigated plants were performed at three time-points throughout berry development (green berry, veraison and harvest). Berry growth and composition were examined during ripening. According to the results, water deficit resulted in reduced berry size and increased levels of soluble sugars, total phenols and anthocyanins. The expression profile of specific genes, known to control grape color, aroma and flavor was altered by water availability during maturation in a cultivar-specific manner. In agreement with the increased concentration of phenolic compounds due to water deficit, genes of the phenylpropanoid pathway in the red-skinned Agiorgitiko exhibited higher expression levels and earlier up-regulation than in the white Assyrtiko. The expression profile of the other genes during maturation or in response to water deficit was depended on the vintage.

## 1. Introduction

Plants are exposed to diverse environmental conditions, including numerous abiotic stresses. Constant changes in climate patterns already threaten global food security with devastating effects in some basic food-producing areas [1]. Models predict that enhanced rainfall humidity followed by abrupt drought periods are among the most dynamic climate changes that jeopardize crop marketability and production [2]. Prolonged drought is decisive for plant growth and yield reduction, affecting crop plant distribution around the world.

Grapevine is one of the well-adapted crops to the South European Mediterranean climate. However, reduction in rainfall and increased evapotranspiration are predicted in the near future due to global warming [2]. The combined effect of drought and high air temperature during the summer regularly inhibits grapevine growth and reduces yield and quality of wine production [3].

Interestingly, in the case of grapevine, moderate water deficit is also known to improve the quality characteristics of grape berries and subsequently the wine composition [4,5]. The reduced vegetative growth leads to improved canopy microclimate [6] and redirects sugars distribution between shoot and ripening berries [7,8]. In addition, the smaller berry size due to transient water deficit results in higher skin to pulp ratio [4,9] and consequently to higher concentration of skin-located aroma and flavor compounds (secondary metabolites) [10]. Most importantly, water deficit is known to cause changes in gene expression and modulate metabolic pathways controlling the accumulation of secondary metabolites that influence berry and wine quality traits [11,12].

Therefore, controlled deficit irrigation is considered a useful strategy in viticultural practices and has been studied in various cultivars and diverse climatic conditions [13,14,15]. Application of water deficit either early (i.e., before veraison) or late in the season results in higher concentration of anthocyanins and phenolic compounds [12,16,17,18]. Further, drought stress causes alterations in the biosynthesis of volatile compounds [18,19,20,21]. However, controlled deficit irrigation studies generated contradictory results regarding berry constituents, mainly because of different irrigation regimes and variations in weather conditions that could lead to decisive fluctuations in water availability [15,19,22]. The variegated response to drought stress is also known to depend on the genetic background of the cultivar [23].

Considering that the overall phenotype, such as grape quality traits, is determined by the combinatorial action of the genotype and the environment, it is fundamental to understand the effect of environment on the gene expression networks that facilitate berry development and quality features. The availability of grapevine genome [24] and high-throughput transcriptomic methods, like microarrays and RNA sequencing, have enriched our knowledge about tissue-specific, development- or environment-controlled gene expression patterns [25]. Specifically, in the case of water deficit, it is known that approximately 13% of grape genes are differentially expressed, mainly in the skin and pulp [10]. Recent transcriptome studies showed that drought alters the expression profiles of genes involved in the aroma and flavor compound biosynthesis in both red- and white-colored cultivars [18,20,26,27].

Grapevine is one of the most important crops in Greece. Over 60,000 ha are covered with winegrapes, consisting of more than 90% domestic cultivars, namely Savatiano, Assyrtiko (white-coloured), Roditis (pink-colored), Agiorgitiko and Xinomavro (red-colored). Foreign cultivars such as Cabernet Sauvignon, Merlot and Syrah are also cultivated. The wider area of Eastern Macedonia and Thrace, where the experimental vineyards are located, is a small vine-growing region in Greece but has exceptional geographical characteristics. The cultivated vineyards in the region account for 4% of the total vineyards of Greece (Hellenic Statistical Authority; http://www.statistics.gr/en/home, accessed on 26 July 2021). Until recently, vine growing was not a basic pillar of local economy and wine production was limited. However, the significant variation in topography combined with the Mediterranean climate qualifies the area for grapevine cultivation and wine production. It is worth mentioning an increase in wine grapes yield from 15,820 to 24,285 tones (i.e., more than 50%) in a five-year period (2013–2018).

Most studies on drought stress conducted under Greek vineyard conditions have focused almost exclusively on the phenolic composition and anthocyanin content of grapes produced by the foreign and domestic red-colored cultivars Cabernet Sauvignon and Syrah [28,29,30,31], and Agiorgitiko and Xinomavro, respectively [19,32,33,34]. Despite the fact that the quality traits of domestic cultivars are of great importance in viticulture and enology, holistic approaches in response to water deficit although challenging, are still lacking.

Herein, we studied the effect of water deficit on gene expression and correlated gene expression levels with the observed berry quality traits, such as phenols and anthocyanins in leading Greek white- and red-colored cultivars such as Assyrtiko and Agiorgitiko, respectively. Further, we also evaluated the expression profile of various genes that contribute to grape aroma and flavor. Our results showed that the deficit irrigation treatment has a strong impact on both grape berry composition and gene expression.

## 2. Results

### 2.1. Climatic Conditions and Vine Water Status

**Figure 1:**
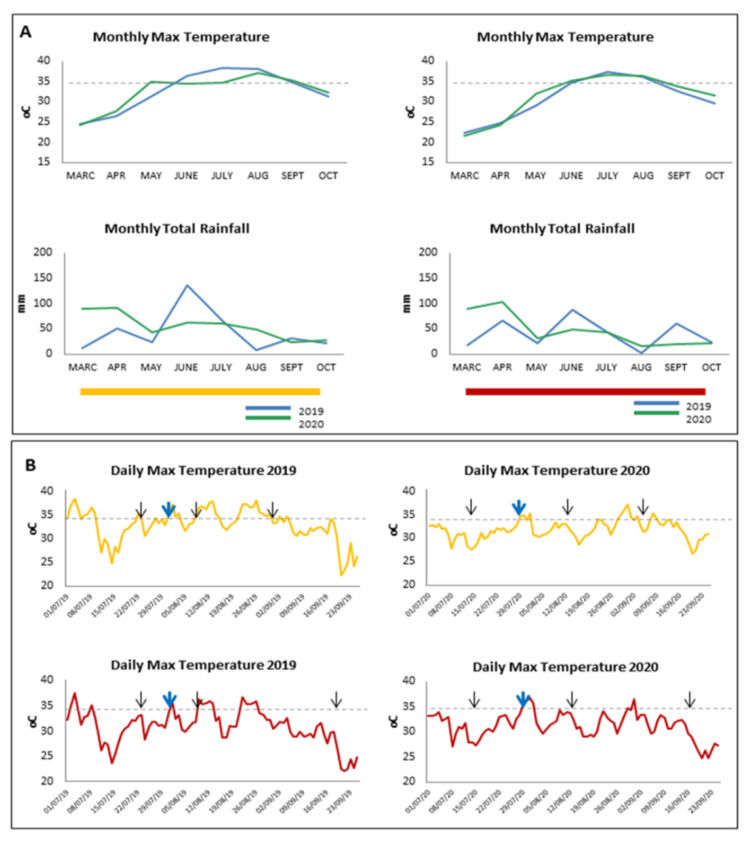

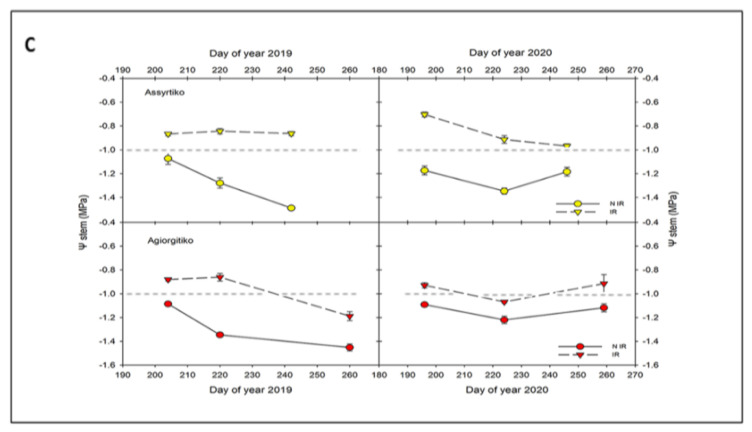
Weather conditions at the experimental vineyards and vine water status (**A**). Evolution of monthly maximum temperature and total rainfall from March to October during the seasons 2019 and 2020; left panel: Drama (Assyrtiko—yellow bar), right panel: Kavala (Agiorgitiko—red bar), blue line: 2019, green line: 2020 (**B**). Evolution of daily maximum temperature during the summer months of 2019 and 2020; upper panel: Drama (Assyrtiko—yellow line), lower panel: Kavala (Agiorgitiko—red line). Black arrows indicate the days of sampling and blue arrows the period of veraison (**C**). Stem water potential (Ψstem) during ripening. Vertical bars indicate the standard deviation of mean values. IR, irrigated; NIR, non-irrigated.

The experimental vineyards are located in neighboring narrow valleys, both at approximately 200 m altitude, in the area of North Greece close to the Aegean Sea (Supplementary Figure S1). The Mediterranean climate of the region is generally characterized by mild winters and temperate summers. During the summer of 2019 (June to August), the total amount of rainfall was higher comparable to 2020 (Figure 1A). At the cultivated location of white-colored Assyrtiko vineyard, the number of days with temperature above 35 during July and August of 2019 was higher than the respective months of 2020. (Figure 1B). As a consequence, the ripening period of Assyrtiko (i.e., veraison to harvest) in 2019 vintage was shorter than 2020. At the vineyard of Agiorgitiko, although the total rainfall in 2019 was higher than 2020, the temperature differences between the two seasons were almost negligible, resulting in ripening periods of approximately equal duration.

Assessment of vine water status was performed through stem water potential (Ψs), a physiological parameter regularly utilized as an accurate indicator [35]. Midday stem water potential was affected by the irrigation regime (Figure 1C). Non-irrigated vines of both cultivars constantly exhibited lower Ψs values (i.e., between −1.1 and −1.4 MPa) in contrast to irrigated vines (−0.8 and −1.0 MPa), in both vintages. According to Ψs critical values [36], drought stress is considered moderate to severe when minimum values are between −1.2 and −1.4 MPa and weak when the range is between −0.6 and −0.9 MPa. In agreement with water potential pattern, other physiological parameters such as stomatal conductance (gs), net assimilation rate (A) and evaporation (E) were also decreased in non-irrigated plants (Supplementary Figure S2A–C). Particularly, stomatal conductance values were recorded between 0.15 and 0.05 mol H_2_O m^−2^ s^−1^, which is considered the threshold of severe water deficit [13]. Taken together, the results show that the non-irrigated vines were under almost severe drought stress in both seasons.

### 2.2. Berry Ripening and Grape Juice Composition

In order to investigate the effect of drought stress on berry quality traits, a number of parameters, such as berry weight, yield, total soluble solids, titratable acidity, total phenols and anthocyanins, were measured at three time-points throughout berry development (green berry, E-L 33; veraison, E-L 36; harvest, E-L 38).

#### 2.2.1. Grape Berry Size, Soluble Sugars and Acidity

**Figure 2:**
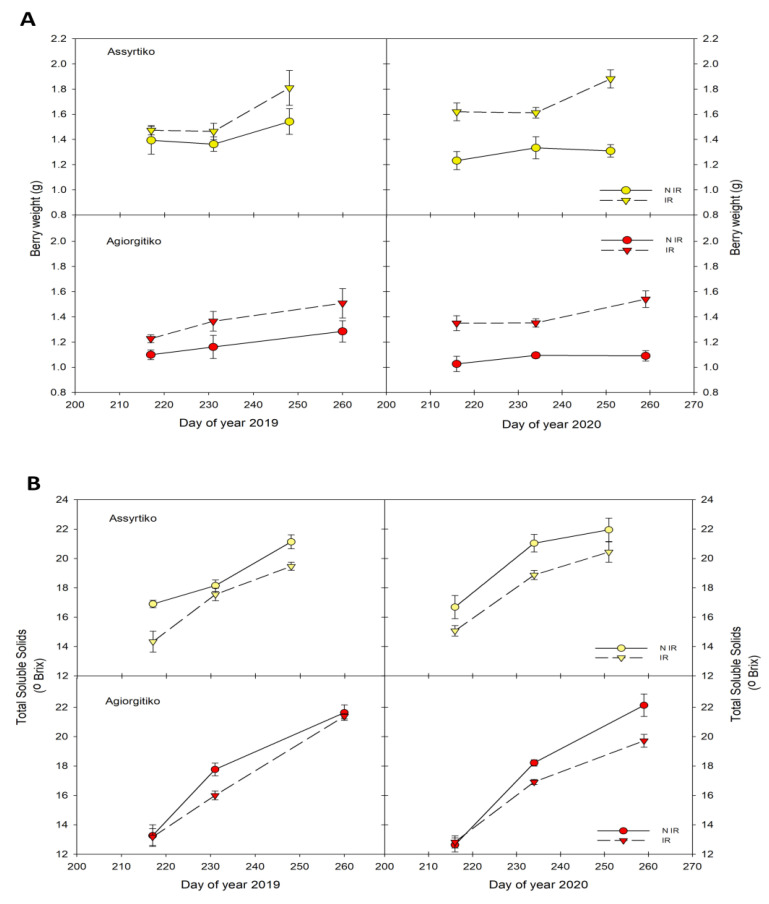

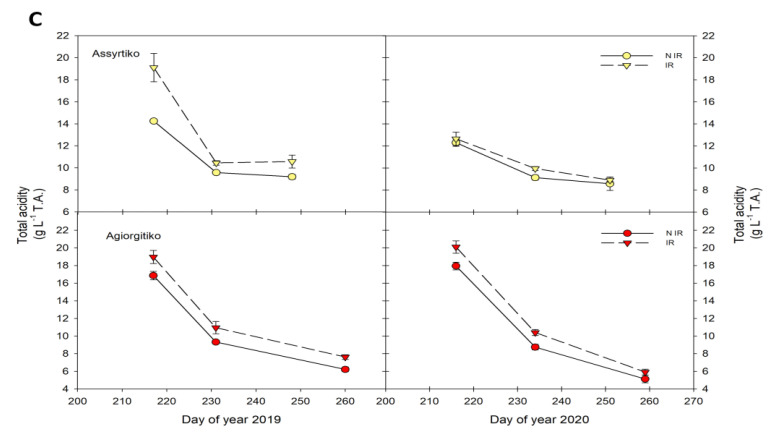
The effect of water deficit on grape berry characters during ripening. (**A**). Berry weight (**B**). Total soluble solids (°Brix). (**C**). Berry titratable acidity. Vertical bars indicate the standard deviation of mean values. IR, irrigated; NIR, non-irrigated.

Berry size was decreased in non-irrigated plants compared to irrigated in all samples, especially during the second vintage 2020 (Figure 2A). It is worth mentioning that the rainfall during the summer months of 2019 was higher than that of 2020 (Figure 1A). In irrigated Assyrtiko vines, berry weight remained constant between the first two samplings and increased rapidly at harvest stage in both vintages, possibly indicating an effect of overripeness. A similar pattern was also observed in Agiorgitiko during 2020 vintage. Moreover, in irrigated vines, grape berries reached a similar maximum size during 2019 and 2020 vintages (approximately 1.8 g for Assyrtiko and 1.5 g for Agiorgitiko). Similarly, in non-irrigated vines of both cultivars, no significant increase in berry weight was observed. Grape berries of non-irrigated plants in 2019 vintage exhibited greater size, especially at harvest stage, than 2020, probably due to increased rainfall the summer months of 2019 (Figure 1A). The results imply that the water availability is a crucial driver of berry size. Remarkably, no significant differences in final yield of irrigated vines between the two vintages were observed at harvest, as indicated by yield/vine and cluster weight (Supplementary Figure S3). However, both parameters were significantly higher in irrigated plants compared to non-irrigated. Interestingly, among non-irrigated plants, yield/vine in Agiorgitiko was significantly higher in 2020 vintage (2.9 compared to 2.4 kg/vine in 2019) and a similar tendency was observed in non-irrigated Assyrtiko plants (Supplementary Figure S3).

Among the most important berry quality traits is the grape juice composition, determined mainly by the total soluble solids (TSS; expressed in °Brix) and the titratable acidity (TA; expressed in tartaric acid g L^−1^). Grape berries in both vintages exhibited a constant increase of total soluble solids during maturation (Figure 2B) and higher concentrations of TSS were detected in non-irrigated vines (Figure 2B).

Specifically in Agiorgitiko, water deficit resulted in higher TSS levels at the end of veraison stage of both years and at harvest of 2020. In Assyrtiko, a noticeable increase of TSS concentrations was observed at veraison of 2020. Remarkably, in irrigated and non-irrigated plants of Assyrtiko TSS levels at middle veraison stage were significantly lower in 2019 compared to 2020.

In contrast to total soluble solids, TA was found to decrease during maturation in both irrigated and non-irrigated plants (Figure 2C). Titratable acidity was not significantly affected by water deficit except Assyrtiko berries of 2019 vintage. While the TA at the green berry stage of all plants was at similar levels, the irrigated Assyrtiko of 2019 vintage exhibited higher TA values than that of 2020 vintage (Figure 2C). Moreover, a statistically significant increase in titratable acidity levels (1.2–1.7 g L^−1^ TA) in both irrigated and non-irrigated plants was observed at harvest stage of 2019 compared to 2020.

#### 2.2.2. Total Anthocyanins and Phenols in Grape Berries

**Figure 3:**
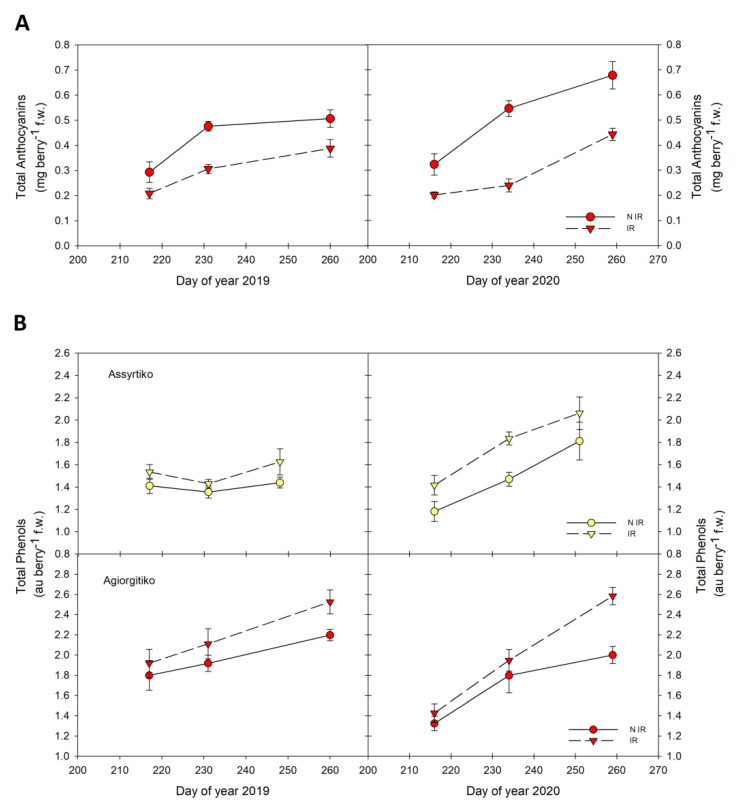
The effect of water deficit on anthocyanins and total phenols during ripening. (**A**). berry total anthocyanins of Agiorgitiko. (**B**). berry total phenols of the two cultivars. Vertical bars indicate the standard deviation of mean values. IR, irrigated; NIR, non-irrigated.

In the red-colored cultivar Agiorgitiko, anthocyanin content per berry was increased during maturation and was higher in non-irrigated compared to irrigated plants (Figure 3A). The increase of anthocyanin levels in non-irrigated plants was rapid until the end of veraison in both vintages, followed by either a slight or a significant increase at harvest of 2019 and 2020, respectively (Figure 3A). The anthocyanin content in non-irrigated plants at harvest stage of 2020 (0.7 mg berry^−1^ f.w.) was significantly higher (*p* < 0.05) than that of 2019 (0.5 mg berry^−1^ f.w.). Even though the difference in anthocyanin levels at harvest in irrigated plants was not statistically significant between the two vintages, a similar trend was also observed.

Interestingly, the total phenolic content of berries exhibited a diverse profile, dependent on the cultivar examined. A gradual increase in total phenols during maturation was observed in Agiorgitiko in both vintages, with significantly higher levels (*p* < 0.05) in non-irrigated compared to irrigated plants (2.5 versus 2.2 au berry^−1^ f.w. in 2019 and 2.6 versus 2 au berry^−1^ f.w. in 2020) at harvest stage (Figure 3B).

On the other hand, no significant changes during maturation of irrigated Assyrtiko were observed in the first year (2019). Remarkably, an increase in total phenolic content was observed the second year (2020), mainly at veraison stage (Figure 3B). Total phenols level in non-irrigated plants of Assyrtiko was significantly higher at veraison and at harvest stages of 2020 (1.8 and 2.1 au berry^−1^ f.w.) compared to that of 2019 (1.4 and 1.6 au berry^−1^ f.w.).

#### 2.2.3. Multivariate Statistics of Chemical and Phenolic Changes upon the Phenological Stages under Field Conditions

To improve the visualization and interpretation of the results and to examine whether the measured variables of each cultivar could distinguish between pre-established groups i.e., drought stress, principal components analysis (PCA) was performed. The previously described berry data were analyzed at the three phenological stages (green berry, veraison and full maturity).

The first two components of PCA score plots could explain 87.1% of the variation in Agiorgitiko, with the first principal component (PC1) accounting for 64% and the second (PC2) for 23.1%, and 80.8% of the variation in Assyrtiko, with the first principal component (PC1) accounting for 58.1% and the second (PC2) for 22.7% (Supplementary Figure S4). Furthermore, unsupervised PCA score plots of the two first components readily discriminated the three stages of berry development (Supplementary Figure S4C). Projection on these two axes clearly separated samples into three groups. Overall, PCA showed that the irrigation treatment effect was stronger than the vintage effect in both cultivars (Supplementary Figure S4A,B).

**Figure 4:**
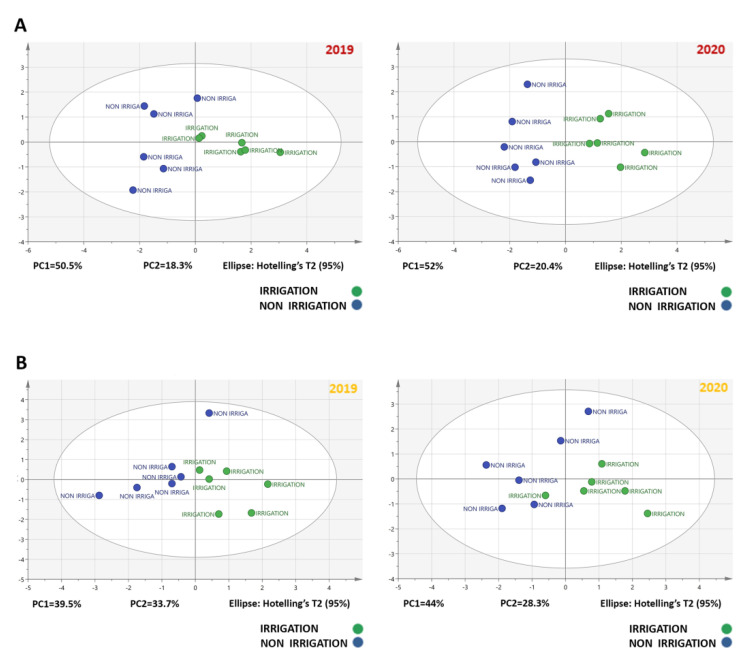
Classification using PCA plot with “irrigation treatment” as depended variable on quality berry characteristics (chemical and anthocyanins- total phenols) data from grape berries cv. Agiorgitico (**A**) and Assyrtiko (**B**) at harvest stage in 2019 and 2020 vintages. Variables in score plots are colored according to the irrigation treatment.

Another unsupervised PCA analysis was performed to show the differences in the chemical and phenolic composition of grape berries at harvest in the two Greek cultivars. The PCA score plot of the two first components explained approximately 70% of the variation for the red-colored cultivar Agiorgitiko during the two vintages, 2019 and 2020, exhibiting a high degree of confidence and an excellent separation of samples with PC1 explaining 50.5% and 52%, and PC2 explaining 18.3% and 20.4% of variance, respectively (Figure 4A). Projection on these two axes clearly separated samples into two groups. The discrimination of these two groups of samples showed a change in the grape berry chemical and phenolic composition according to irrigation. A similar trend was also observed in the white-colored cultivar Assyrtiko during the harvest stage in two vintages, 2019 and 2020, with the first principal component (PC1) accounting for 39.5% and 44% and the second (PC2) for 33.7% and 28.3%, respectively (Figure 4B).

### 2.3. Gene Expression in Grape Berries

Recent results have highlighted that drought cause alterations in grape berry transcriptome [18,20,27]. In the present study, the effect of water deficit on gene expression was examined by RT-qPCR analysis of berry samples collected at three different stages (green berry, veraison and harvest) during the 2019 and 2020 vintages. A targeted expression analysis was performed on key genes from metabolic pathways known to contribute to grape color, aroma and flavor. Assuming that most of these genes exhibit skin-specific or skin-preferential expression [10], we used skin tissue samples to isolate RNA. We analyzed the expression of phenylalanine ammonia lyase (VviPAL; VIT_13s0019g04460) and *trans*-cinnamate 4-monooxygenase (VviC4H; VIT_06s0004g08150) genes that are involved in the initial steps of the phenylpropanoid pathway [37] as well as UDP-glucose-flavonoid 3-O-glycosyltransferase (VviUFGT; VIT_04s0023g01290) gene that encodes for the rate-limiting enzyme of anthocyanin biosynthesis [38]. Furthermore, we investigated the expression profiles of 1-deoxy-D-xylulose-5-phosphate synthase (VviDXS; VIT_05s0020g02130) and carotenoid cleavage dioxygenase (VviCCDD1; VIT_13s0064g00840) genes regulating the first steps of terpenoid [39] and norisoprenoid [40] pathways, respectively. We also examined lipoxygenase A (VviLOXA; VIT_06s0004g01450) gene involved in biosynthesis of volatile compounds via fatty acid metabolism [41] and finally, γ-glutamyl-transpeptidase (VviGGT; VIT_11s0016g02830) gene related to volatile sulphur compounds biosynthesis [42] (Supplementary Figure S5).

#### 2.3.1. Genes of the Phenylpropanoid Pathway

**Figure 5:**
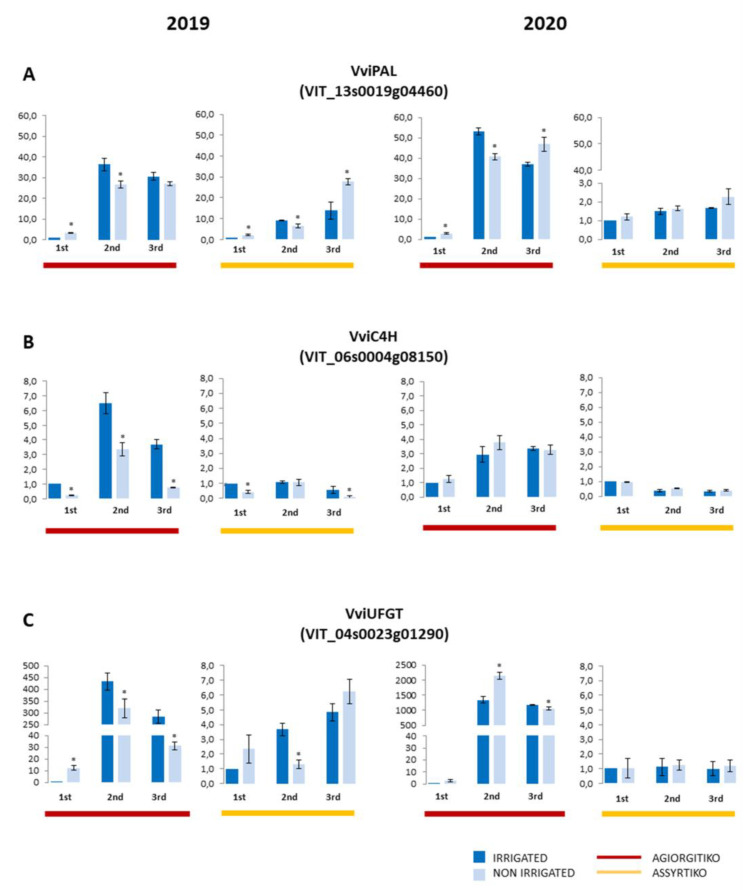
Expression level of genes involved in phenylpropanoid pathway (VviPAL (**A**), VviC4H (**B**) and VviUFGT (**C**)) in Agiorgitiko (red bars) and Assyrtiko (yellow bars) during the two seasons of the experiment (2019 and 2020). The expression levels of irrigated plants are shown in dark blue and those under water deficit are shown in light blue. Vertical bars represent the standard deviation and asterisks indicate the statistically significant differences (Student’s *t*-test, * *p*-Value < 0.05) between irrigated and non-irrigated plants of the same sampling period. The three samples (green berry, 1st; veraison, 2nd and harvest, 3rd) are indicated under each pair of graphs.

We initially investigated the expression profile of genes that encode enzymes of the phenylpropanoid pathway that leads to biosynthesis of phenolic compounds and anthocyanins. The expression analysis of VviPAL gene in irrigated plants of red-colored cultivar Agiorgitiko showed a significant up-regulation after veraison, followed by a slight decrease at harvest stage in both vintages (Figure 5A). Although the relative expression was obviously lower in white-colored Assyrtiko, there was also a constant increase during maturation in 2019, while almost negligible changes were observed in 2020 (Figure 5A). The expression of VviC4H gene was lower than VviPAL expression and had almost similar pattern of accumulation during the berry maturation stages of Agiorgitiko. However the expression of the same gene in Assyrtiko remained at similar levels or was slightly decreased at the last stages of ripening (Figure 5B).

Water deficit resulted in increased VviPAL expression compared to controls in both cultivars at green berry and at harvest, with the exception of 2019 harvest samples of Agiorgitiko. However, reduced expression was observed at middle veraison in both cultivars in 2019 vintage and in Agiorgitiko in 2020 when compared to control plants. The VviC4H mRNA levels exhibited a similar pattern of accumulation under drought stress except in 2019 vintage (Figure 5B). As in the case of irrigated plants, the VviC4H gene expression pattern of non-irrigated Assyrtiko was similarly reduced during maturation. In non-irrigated plants of both cultivars, the expression level of VviC4H was lower in green berry and harvest of 2019 (Figure 5B).

The VviUFGT gene encoding for the critical step in anthocyanin biosynthesis, exhibited a dramatical up-regulation after veraison and an expression profile almost similar to VviPAL in Agiorgitiko (Figure 5C). We uncovered that the expression levels of VviUFGT gene were far lower in Assyrtiko than Agiorgitiko, a gradual increase was only observed in 2019 samples. In non-irrigated plants of Agiorgitiko, VviUFGT expression was high at green berry stage of 2019 while it was decreased at harvest of both vintages when compared to irrigated Agiorgitiko plants (Figure 5C). Interestingly, reduced expression levels compared to irrigated plants were observed at veraison samples of 2019 but in contrast, the expression was higher than in controls at the same stage of 2020. On the other hand, no significant changes between irrigated and non-irrigated plants were observed in Assyrtiko throughout ripening in both vintages, except for veraison of 2019 (Figure 5C).

Taking together the results of the expression of the three genes involved in phenylpropanoid pathway, we could delineate that their transcript levels exhibited an up-regulation at veraison followed by a decline towards harvest in Agiorgitiko and were obviously higher than in the white-skinned Assyrtiko.

#### 2.3.2. Other Genes Related to Aroma Compounds

**Figure 6:**
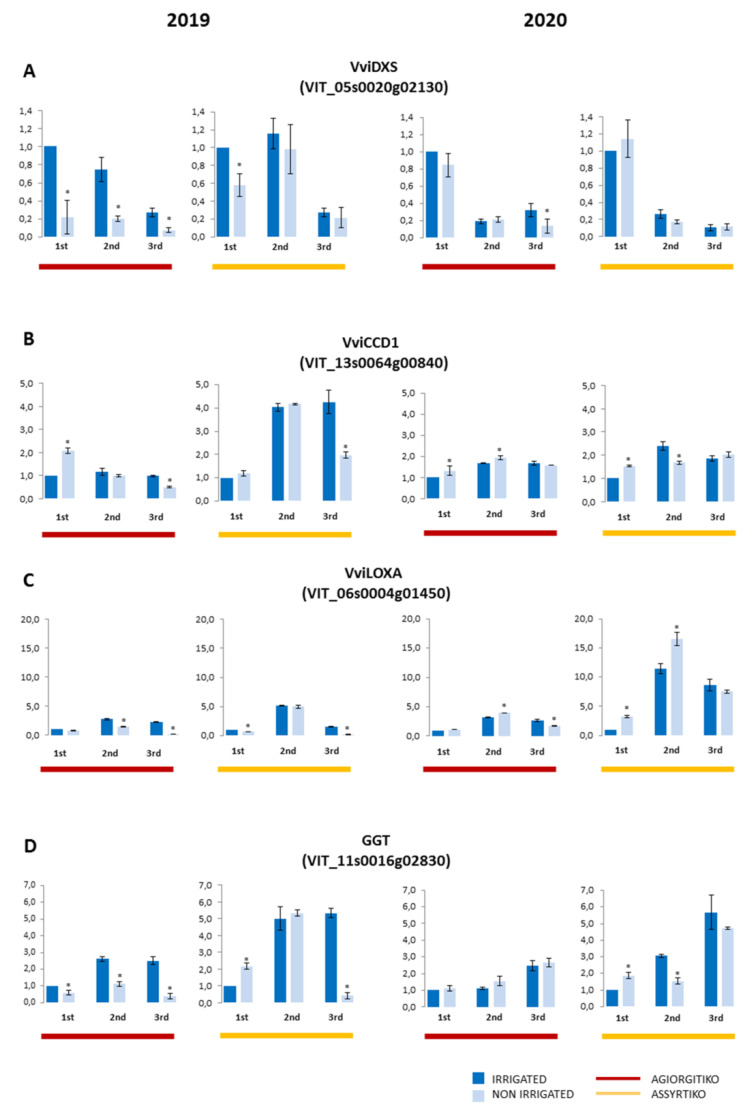
Expression level of genes involved in the biosynthesis of various aroma compounds (VviDXS (**A**), VviCCD1 (**B**), VviLOXA (**C**) and VviGGT (**D**) in Agiorgitiko (red bars) and Asyrtiko (yellow bars) during the two seasons of the experiment (2019 and 2020). The expression levels of irrigated plants are shown in dark blue and those under water deficit are shown in light blue. Vertical bars represent the standard deviation and asterisks indicate the statistically significant differences (Student’s *t*-test, * *p*-Value < 0.05) between irrigated and non-irrigated plants of the same sampling period. The three samplings (green berry, 1st; veraison, 2nd and harvest, 3rd) are indicated with numbers under each pair of graphs.

In addition to the phenylpropanoid pathway, we examined the expression patterns of genes belonging to biosynthetic pathways of volatile compounds that contribute to the aroma potential of grape berries. The expression of VviDXS gene that encodes for the enzyme catalyzing the initial step in terpenoid biosynthesis (Supplementary Figure S5) showed a declining trend during berry maturation (Figure 6A). Water deficit caused a decreased VviDXS expression at green berry stage in 2019 of both cultivars and throughout the maturation berry stages of Agiorgitiko. However, no significant difference to controls was observed in the following year (Figure 6A). On the other hand, the VviCCD1 gene encoding for the key enzyme in norisoprenoid pathway (Supplementary Figure S5), exhibited a different profile with constantly increased expression during maturation in Assyrtiko in both vintages (mainly in 2019), while in Agiorgitiko an increase was observed only in 2020 (Figure 6B). Water shortage resulted in up-regulation of VviCCD1 gene in both cultivars at green berry stage of both years, but a decline at harvest stage of 2019 was observed. All together the results indicate a different response of the genes controlling terpene and norisoprenoid biosynthesis to water deficit.

The lipoxygenase gene examined in this study, exhibited an almost similar expression profile in both cultivars showing an up-regulation after veraison, followed by a slight decrease at harvest stage (Figure 6C) which coincides with the decrease of vegetal flavors towards harvest. Additionally, the accumulation of LOXA mRNA in Assyrtiko was obviously higher in 2020 samples than that of 2019. Interestingly, in non-irrigated plants of Assyrtiko, VviLOXA expression was decreased compared to the controls in green berry samples of 2019, but it was increased in the corresponding samples of the next year. Similarly, changes in LOXA expression between the two vintages were also observed at veraison stage in non-irrigated plants of Agiorgitiko. The increase observed at veraison stage in both irrigated and non-irrigated cultivars was followed by reduction at harvest stage (Figure 6C). Finally, the expression analysis of the γ-glutamyl-transpeptidase gene VviGGT exhibited an up-regulation during the progression of berry maturation in irrigated plants of both cultivars (Figure 6D). Water deficit resulted in down-regulation of the VviGGT gene in Agiorgitiko samples of 2019 but almost no differences in the expression level were detected when compared to irrigated plants the next year. In non-irrigated plants of Assyrtiko the expression of the VviGGT gene had a distinctively opposite pattern at veraison and mature stages (Figure 6D).

## 3. Discussion

Grapevine is considered to be a drought-resilient species, traditionally covering semiarid areas. However, environmental factors, such as the climatic variability, influence grape and wine quality. The different weather conditions among the two years influenced the maturation processes of both cultivars and therefore, the vintage effect was evident in the grape parameters examined. For instance, grape berries of non-irrigated plants in 2019 vintage exhibited greater size than 2020, probably due to increased rainfall during the summer months of that year. Vintage-dependent variations were also observed in grape juice components. Total soluble solids levels were decreased at veraison stage of 2019 in both irrigated and non-irrigated plants of Assyrtiko indicating the negative effect of high temperatures on sugars accumulation, as has been already reported for other cultivars [43,44]. Although total soluble solids and titratable acidity are important grape berry parameters, an appropriate sugar/acid maturity is insufficient to ensure the quality of grapes and therefore, the quality of the wine obtained. The most important factor affecting wine quality is the phenolic content of grapes at harvest, considering that a number of sensory attributes of wine are directly associated with phenolic compounds [45]. Several studies have demonstrated that daytime temperatures could affect the accumulation and composition of phenolic compounds in grape berries [46,47,48]. At harvest of 2020 total phenols in non-irrigated vines of Assyrtiko was higher compared to that of 2019, suggesting that the high temperatures during the summer of 2019 could have a negative effect in total phenolic content.

Principal components analysis showed that the irrigation treatment effect had a strong impact on grape berry parameters of both cultivars. According to the vine physiology parameters, such as stem water potential and stomatal conductance, the non-irrigated plants of both cultivars were under almost severe drought stress (i.e., Ψs values constantly below −1.1 MPa and gs values below 0.15 mol H_2_O m^−2^ s^−1^), regardless the vintage. Water deficit resulted in reduced berry size and increased levels of total soluble solids and total phenols in both cultivars and in anthocyanins levels in the red-colored cultivar Agiorgitiko. However, at harvest, a statistically significant increase in the phenolic content in non-irrigated vines was observed only in Agiorgitiko. Considering that higher concentrations of grape juice components under drought stress depends on a decrease in berry size together with transcriptional changes [11,49], it was challenging to investigate the expression profile of genes involved in phenolic compounds biosynthesis.

Recently accumulated results from transcriptomic studies have shown that phenylpropanoid pathway genes were differentially expressed under water deficit [18,26,27]. Herein, we showed that the expression profile of the genes examined was clearly different between the two cultivars. The expression levels of VviPAL and VviC4H were obviously higher in the red-colored Agiorgitiko than in the white-colored Assyrtiko, which concurred with the difference in total phenolic content between the two cultivars. During berry development in Agiorgitiko irrigated plants, the expression of these two genes, as well as VviUFGT, was significantly increased at veraison and then slightly declined towards harvest in both vintages. This expression profile has been also observed in other red-color cultivars like Norton and Merlot [50,51]. Taken together, these results suggest that the expression of phenylpropanoid pathway genes is developmentally regulated in at least red-colored cultivars. A similar expression pattern of these genes during maturation implies that a coordinated regulation could exist as it has been suggested for Norton and Cabernet Sauvignon berries [26,50]. Drought stress resulted in early up-regulation of VviPAL expression at green berry stage in Agiorgitiko, contributing to the increased phenolic compounds accumulation in non-irrigated plants. Water deficit has been already reported to increase expression levels of phenylpropanoid pathway genes in red-colored cultivars [10,12,26,27]. Similarly, drought caused increased expression of VviPAL in Assyrtiko at the onset of veraison and at harvest, as has been also reported in white-colored cultivar Tocai Friulano [18]. Nevertheless, this up-regulation was not sufficient to increase total phenol levels when compared to irrigated plants.

A critical step for anthocyanin synthesis is catalyzed by the enzyme encoded by VviUFGT gene. The pattern of VviUFGT mRNA accumulation was similar to the expression profile of VviPAL and VviC4H during berry maturation in Agiorgitiko. Considering that anthocyanin biosynthesis determines the red color of grape berry, the expression was dramatically reduced in the white-colored Assyrtiko. In non-irrigated plants of Agiorgitiko a noticeable up-regulation of VviUFGT was observed at green berry stage of 2019 and at veraison of 2020. Insignificant changes were observed in non-irrigated plants of Assyrtiko in both seasons. Similar expression patterns under water deficit were observed by transcriptomic analysis in the red-colored cultivars Cabernet Sauvignon [11,12,26] and Merlot [17] and the white-colored cultivars Chardonnay [12] and Tocai Friuliano [18]. The weather conditions during the maturation period of 2019 were probably the decisive parameter in modulating VviUFGT expression levels at veraison in non-irrigated plants compared to irrigated Agiorgitiko vines. However, the apparent (approximately ten-fold) up-regulation of VviUFGT gene expression at the green berry stage could be sufficient to increase anthocyanin accumulation observed in non-irrigated plants. In 2020 vintage, VviUFGT expression level at veraison was higher in non-irrigated Agiorgitiko vines compared to irrigated, concurring with the anthocyanin levels.

Apart from phenolic and anthocyanin content, grape and wine quality is affected by volatile organic compounds such as terpenes, C_13_ norisoprenoids, aldehydes and thiols. We selected genes engaged in initial steps of terpenes and C_13_ norisoprenoids biosynthesis, the gene that encodes for the enzyme that catalyzes the first step of the lipoxygenase pathway leading to volatile aldehydes synthesis and a γ-glutamyl-transpeptidase gene related to the subsequent production of sulphur volatile compounds. VviDXS expression was decreased towards harvest in both cultivars as has been also reported in other studies [20,21] while the expression of VviCCD1 was almost constant in Agiorgitiko through maturation and increased after veraison in Assyrtiko. Considering that VviCCD1 catalyzes the cleavage of carotenoids to form C_13_ norisoprenoids [40], these variations could be related to different carotenoid/norisoprenoid ratios in each cultivar. Although in different way (i.e., down-regulation of VviDXS, and constant or increased levels of VviCCD1), the expression profile during maturation suggests that both genes are also developmentally regulated. Nevertheless, the different responses of the two genes to drought were related to the cultivar and the vintage. Water deficit resulted in down-regulation of VviDXS at harvest of both vintages in Agiorgitiko and at green berry stage of 2019 in Assyrtiko. Similar results have been reported for Cabernet Sauvignon [10] and Tocai Friulano [18]. In non-irrigated plants of both cultivars, VviCCD1 expression was increased at the onset of veraison, a response to drought that has been observed in Cabernet Sauvignon but not in Chardonnay [12]. Considering the accumulation of norisoprenoids under water deficit conditions reported in previous studies [19,20,52,53], it seems that norisoprenoid biosynthesis is resilient to drought stress.

The expression of the lipoxygenase VviLOXA gene was increased after veraison and decreased slightly at harvest in both cultivars. Up-regulation of VviLOXA around veraison has been also reported in white-colored Sauvignon blanc berries [42]. No vintage effect was observed in VviLOXA expression in Agiorgitiko but in contrary, the expression levels in Assyrtiko were obviously higher in 2020. Lipoxygenase genes found to be up-regulated by drought stress in both Cabernet Sauvignon and Chardonnay throughout berry development [12]. We observed a similar pattern only the second vintage at the first two phenological stages. An increase in VviGGT expression towards harvest was observed in irrigated plants of both cultivars. Similar expression profile during maturation has been reported in Sauvignon blanc [54]. However, water deficit caused up-regulation of VviGGT expression at green berry stage in Assyrtiko, suggesting an earlier response to drought, while in Agiorgitiko the effect was dependent on the vintage.

The effect of drought on berry development and metabolite profile has been substantially studied producing diverse results. Although the general trend is an increase in phenolic compounds as it was herein observed in the red-colored cultivar Agiorgitiko, and a reduction in terpenes, this response might not be common to all varieties (reviewed in [23]). This variability will be obviously reflected by the expression of corresponding genes. Our results revealed gene expression profiles that were developmentally regulated during maturation regardless the vintage, especially in Agiorgitiko (VviPAL, VviUFGT, VviDXS and VviLOXA). In contrary, most gene expression profiles in white-colored Assyrtiko were significantly affected by the vintage. Water deficit caused early up-regulation of genes at green berry stage (e.g., VviPAL in Agiorgitiko and VviGGT in Assyrtiko), which was observed at forthcoming developmental stages in irrigated plants. More in-depth and wider studies of cultivars in response to environmental stress are important to comprehend grapevine adaptation to arid climates, a critical step towards an optimal choice of irrigation strategy.

## 4. Materials and Methods

### 4.1. Vineyard Site and Experimental Design

All experiments were conducted throughout the 2019 and 2020 growing seasons in two different commercial vineyards, located in Drama (41°12′03″ N, 23°57′10″ E, at elevation 211 m) and Kavala (40°48′43″ N, 23°59′25″ E, at elevation 231 m) in Northern Greece, planted with varieties (*Vitis vinifera* L.) cv. Assyrtiko and Agiorgitiko, respectively. Both vineyards were 12-year-old and grafted on 1103 Paulsen (*V. rupestris* × *V. berlandieri*) at a density of 3333 vines/ha (2.5 m between rows × 1.2 m within rows). Vines were trained on a vertical trellis with three fixed wires and spur-pruned on a bilateral cordon system to a standard 16 nodes per vine. Pest and canopy management, and fertilization were applied according to standard local viticultural practices. The field trial was carried out in three replicated vineyard blocks.

Starting at berry set (E-L 27) through harvest, two water regimes were applied on a weekly basis: irrigation at 100% of crop evapotranspiration ETc (IR) and non- irrigated (NIR). Each treatment was replicated three times in randomized blocks, with 10 consecutive plants for each replication. The water quantities were applied by a drip irrigation system equipped with 4 L/h drip emitters. ETc was estimated from the potential evapotranspiration data (calculated by the Penman-Monteith method) obtained from an on-site automatic weather station (iMETOS, Pessl Instruments GmbH, Weiz, Austria). Water was supplied on either side of the trunk, positioned at 50 cm intervals along the pipe. For IR treatments at Assyrtiko, total water supply was 384 mm in 2019 and 422 mm in 2020, while for Agiorgitiko was 413 mm in 2019 and 438 mm in 2020.

### 4.2. Vine Parameters

Stem water potential (Ψstem) measurements were conducted with the use of a pressure chamber, according to Choné et al. [40]. In each measurement set, three mature leaves of the inside part of the canopy were enclosed in plastic bags and covered with aluminium foil for at least 90 min before measurement, to allow equilibration of Ψs. Measurement of Ψs were performed at midday (12 h 30 to 13 h 30), on three cloudless days per season, corresponding to the growth stages of bunch closure (E-L 33, approximately 20 days after the beginning of irrigation), middle ripening (E-L 36) and harvest (E-L 38). Only the central 4 vines of each replication were used for measurements.

Net assimilation rate (A), stomatal conductance (gs) and evaporation (E) were recorded at midday, simultaneously with Ψs measurements, using the LCi portable gas exchange system (ADC BioScientific Ltd., Hoddesdon, UK). Measurements were taken on three fully expanded, recently matured, sun-lit leaves per plot (photosynthetic photon flux density > 1200 μmol m^−2^ s^−1^) and adjacent to those used for Ψs determination.

### 4.3. Berry Sampling and Must Analysis

Grapes were harvested on 30 August 2019, and 2 September 2020, for Assyrtiko and on 17 September 2019, and 15 September 2020, for Agiorgitiko, from the four chosen vines in each plot. Total yield per plant (kg/vine) and average cluster weight (g) was estimated. Three samplings took place after veraison in 2019 (Day Of Year; DOY) 217, 231, 242 for Assyrtiko and 217, 231, 260 for Agiorgitiko and in 2020 (DOY) 216, 234, 246 for Assyrtiko and 216, 234, 259 for Agiorgitiko. Samples of 200 berries were collected randomly from each plot per sampling date and berry fresh weight was determined. The remaining berries per plot were pressed, and the must was analyzed for soluble solids (°Brix) by refractometry, titratable acidity (g L^−1^ tartaric acid) and pH.

### 4.4. Phenolic Content of Whole Berries

Berries (about 150) from each replicate were homogenized using an Ultra Turrax T25 (IKA-Werke, Staufen, Germany) at 24,000 rpm for 1 min. Total phenol and anthocyanin content was measured according to Iland et al. [55]. Briefly, 1 g of the homogenate was transferred into a centrifuge tube and mixed with 10 mL 50% *v*/*v* aqueous ethanol (pH 2.0) for 1 h. After centrifugation at 3500 rpm for 10 min, 0.5 mL of the supernatant was added to 10 mL 1N HCl and mixed thoroughly for 3 h, then absorbance at 520 nm and 280 nm was recorded. All analyses were performed in triplicates.

### 4.5. RNA Extraction and Gene Expression Analysis by RT-qPCR

Three samplings took place simultaneously with vine physiology measurements in 2019 (DOY) 204, 220 for both, 242 for Assyrtiko and 260 for Agiorgitiko and in 2020 (DOY) 196, 224 for both, 246 for Assyrtiko and 259 for Agiorgitiko. Skins of 10 berries per plot were removed by hand from the grapes, covered with aluminium foil and placed on dry ice. Tissue samples were ground to powder with liquid nitrogen and RNA was extracted by the method of Reid et al. [56]. Briefly, approximately 1 g of ground tissue (berry skin) was extracted with buffer containing 300 mM Tris-HCl (pH 8.0), 25 mM EDTA, 2 M NaCl, 2% (*w*/*v*) CTAB, 2% (*w*/*v*) PVPP, 0.05% (*w*/*v*) spermine at 65 °C for 15 min, mixed thoroughly with equal volume of chloroform:isoamyl alcohol (24:1) and centrifuged. The chloroform:isoamyl alcohol step was repeated and the aqueous phase was collected. The RNA was precipitated with 0.6 volumes isopropanol and 0.1 volumes 3 M sodium acetate at −20 °C overnight, centrifuged and finally dissolved in 100 μL ddH_2_O. RNA samples were treated with DNAse I (Takara Bio, Shiga, Japan) and further purified using phenol:chloroform:isoamyl alcohol (25:24:1) followed by ethanol precipitation. The RNA quantity and quality were determined using a NanoDrop ND-1000 Spectrophotometer (Thermo Fisher Scientific Inc., Wilmington, DE, USA) and verified by 0.8% agarose gel electrophoresis, respectively. Reverse transcription was performed with 2 μg RNA using SMART MMLV-Reverse Transcriptase (Takara Bio) and oligo (dT) primer (Eurofins Genomics, Ebersberg, Germany). The synthesized cDNA was five-fold diluted and PCR conditions were optimized for primers corresponding to selected genes (listed in Supplementary Table S1). The samples were further diluted, and quantitative PCR reactions were performed in the PikoReal Real-Time PCR System (Thermo Fisher Scientific, Vantaa, Finland) using SYBR Select Master Mix (Applied Biosystems, Carlsbad, CA, USA) and applying the following cycler conditions: 2 min at 50 °C, 2 min at 95 °C, followed by 40 cycles of 15 s at 95 °C, 30 s at 62 °C, 30 s at 72 °C. All quantitative PCR reactions were performed in triplicates and melting curve analysis was performed at the end of each reaction to confirm primer specificity. Quantification of gene expression was performed according to the 2^−ΔΔCt^ method [57] and elongation factor 1a (VviEF1a; VIT_06s0004g03240), was used as the reference gene for data normalization.

### 4.6. Statistical Analysis

Principal component analysis (PCA) was used in order to visualize differences and similarities among samples as well as to confirm robustness and analytical variability. PCA was performed on the chemical and on phenolic data of the grape berries using the Umetrics SIMCA^®^ 14.1 software (Sartorius Stedim Data Analytics AB, Umeå, Sweden). Data were subjected to one-way analysis of variance (ANOVA) and Student’s *t*-test was used to assess the statistically significant differences among the mean values.

## Data Availability

Not applicable.

## Supplementary Materials

The following materials are available online at https://www.mdpi.com/article/10.3390/plants10091947/s1, Table S1: List of primers used in RT-qPCR analysis, Figure S1: Map of Northern Greece showing the locations of the experimental vineyards, Figure S2: Grapevine physiological parameters during the seasons 2019 and 2020, Figure S3: The effect of water deficit on grapevine yield during the seasons 2019 and 2020, Figure S4: Classification using PCA plot on berry quality characteristics, Figure S5: Biosynthetic pathways of grape color and aroma compounds.
