# Supplementary material for: The Effect of Water Deficit on Two Greek Vitis vinifera L. Cultivars: Physiology, Grape Composition and Gene Expression during Berry Development
Source: Plants (Basel). 2021 Sep 18;10(9):1947. doi: 10.3390/plants10091947 (PMC8470430; doi:10.3390/plants10091947)
Supplement: Supplementary file 1 [file plants-10-01947-s001.zip › plants-1376485-supplementary/Suppl. Material_1_Final.pdf]

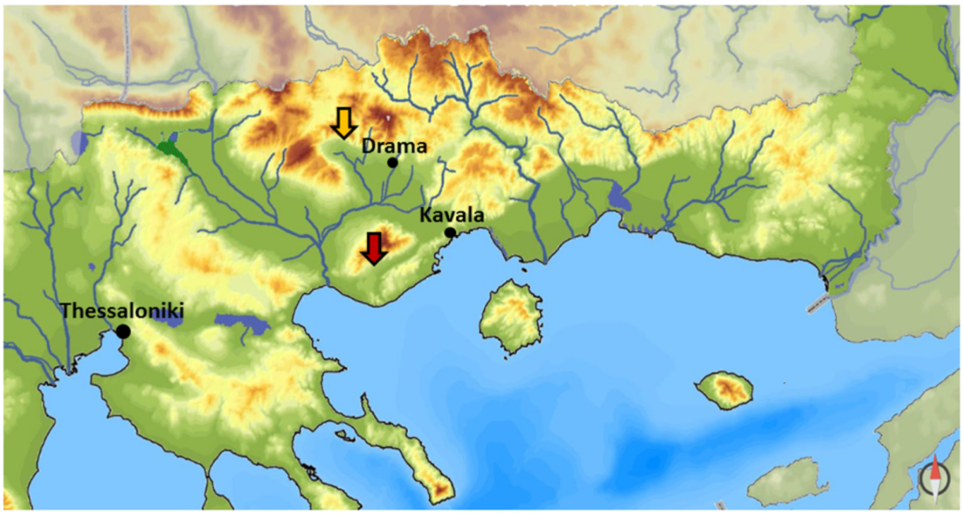

Suppl. Figure S1. Map of Northern Greece showing the locations of the experimental vineyards of Agiorgitiko (red arrow) and Asssyrtiko (yellow arrow).

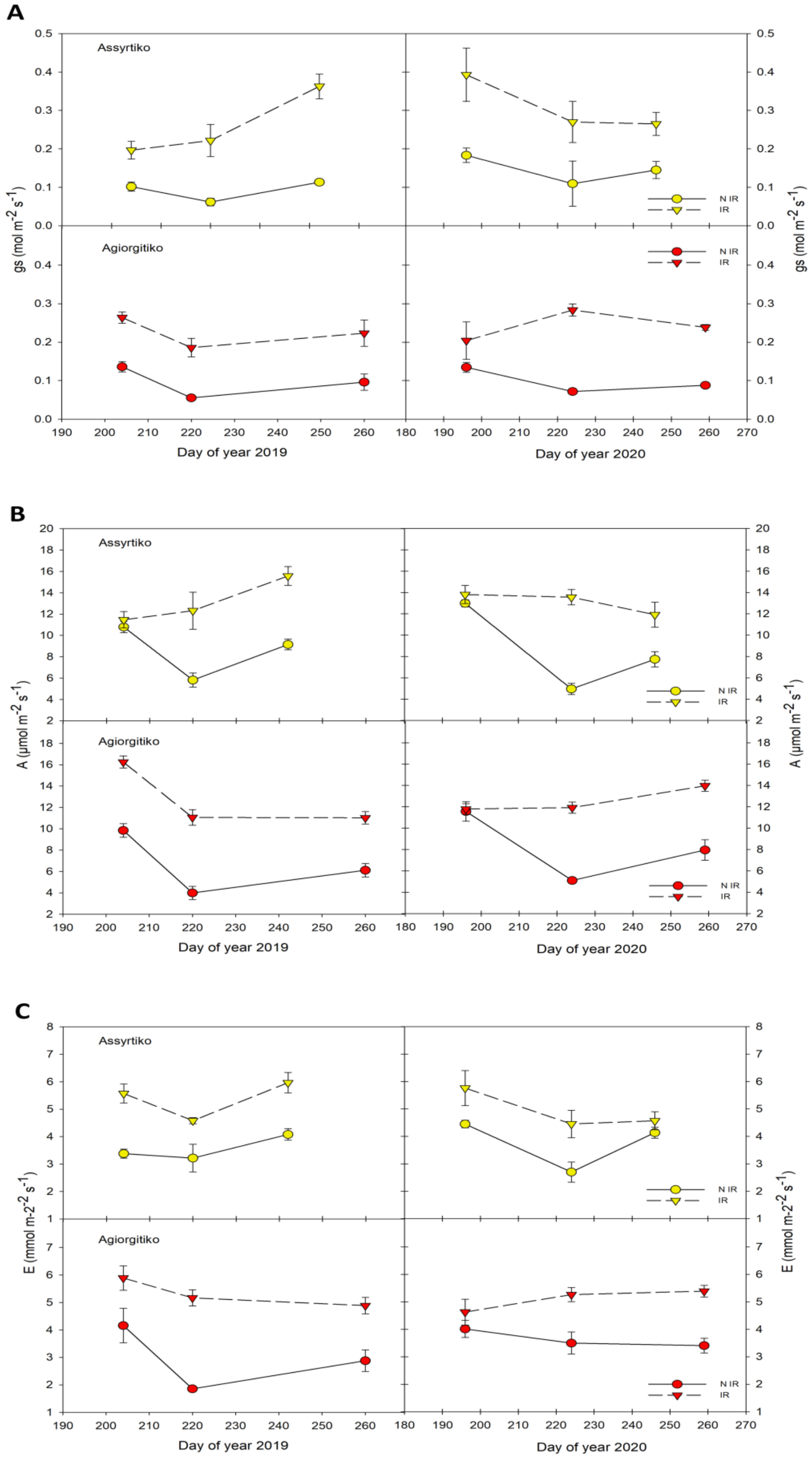

Suppl. Figure S 2. The grapevine physiological parameters during ripening. **A**. stomatal conductance (gs) **B**. net assimilation rate (A), **C**. evaporation (E). Vertical bars indicate the standard deviation of mean values. IR, irrigated; NIR, non-irrigated.

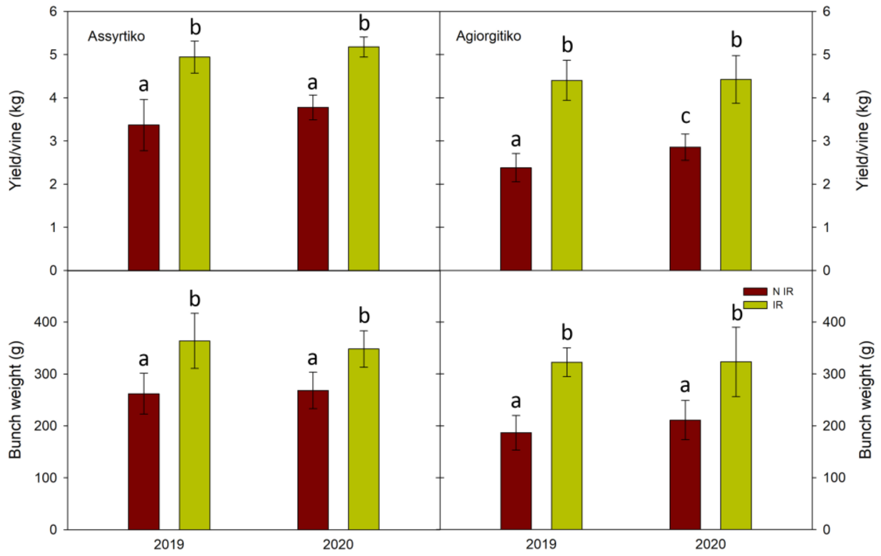

Suppl. Figure S3. The effect of water deficit on grapevine yield. Total yield per vine and bunch weight are shown. Vertical bars represent the standard deviation and different letters indicate statistical significant difference (Student's t-test, p value < 0.05) between irrigated and non-irrigated plants of the same sampling period. IR, irrigated; NIR, non-irrigated.

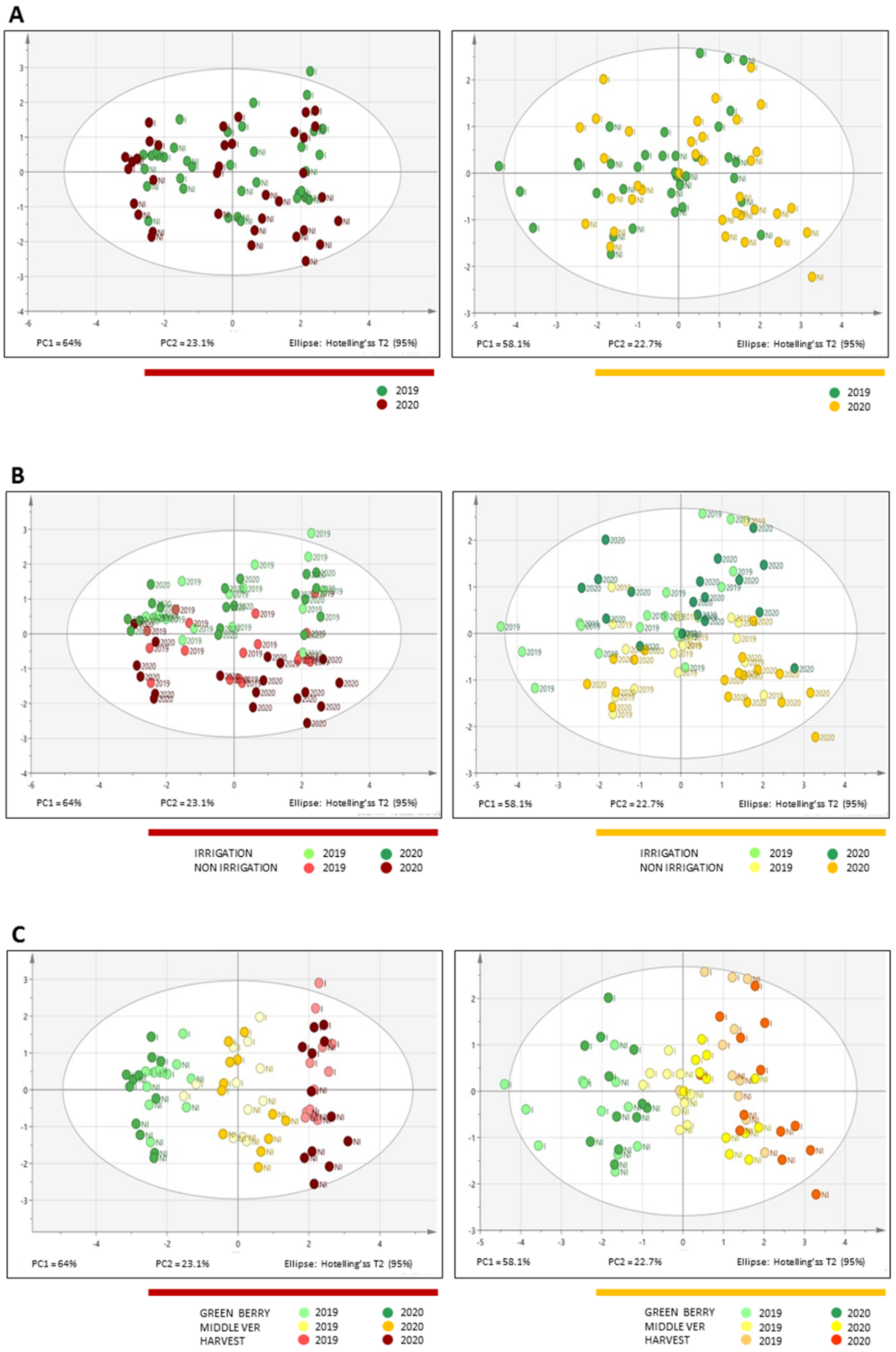

Suppl. Figure S 4. Classification using PCA plot on berry quality characteristics (chemical and anthocyanins- total phenols) data from grape berries cv. Agiorgitiko (red bars) and cv. Assyrtiko (yellow bars) at the three sampling stages (green berry, middle veraison and harvest) in the vintages of 2019 and 2020 considering "vintage" (**A**), "irrigation treatment" (**B**) and "phenological stage" (**C**) as the dependent variable. Variables in score plots are colored according to the vintage, irrigation treatment and phenological stage, respectively. I, irrigated; NI, non-irrigated.

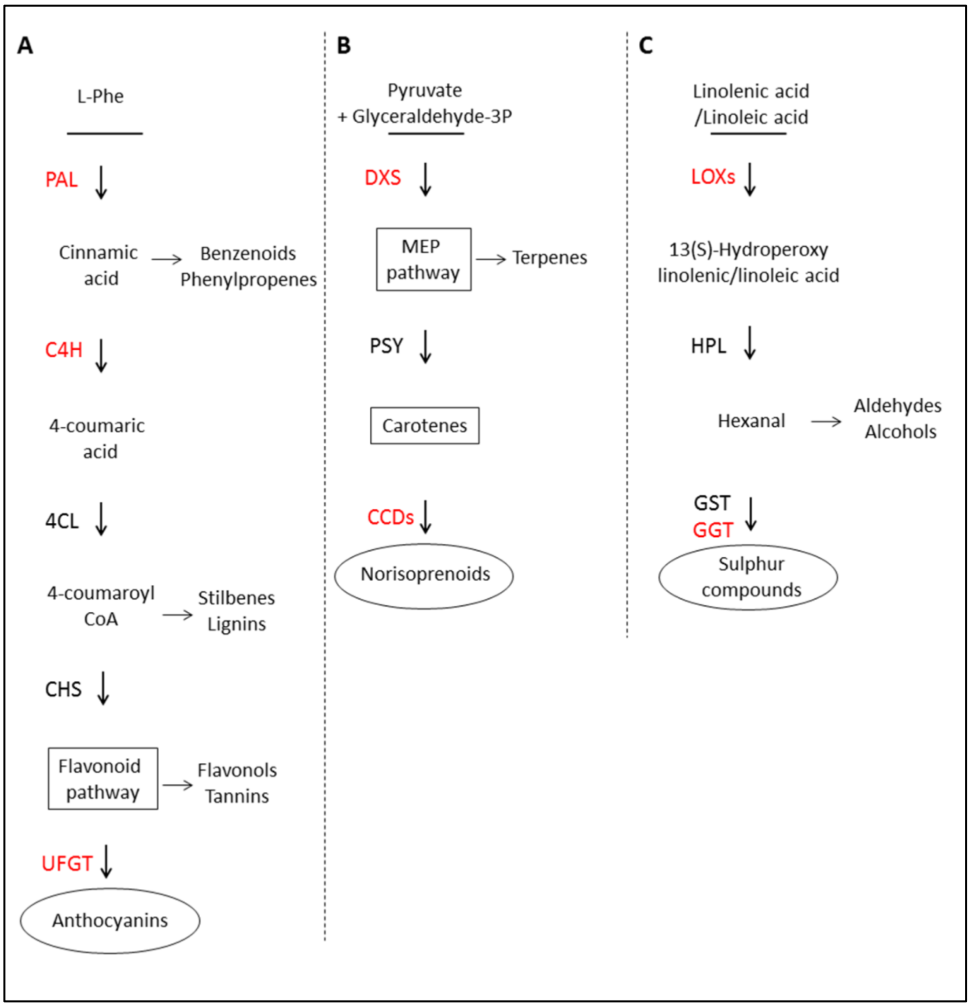

Suppl. Figure S 5. Biosynthetic pathways of grape color and aroma compounds. **A**. the phenylpropanoid pathway that leads to the production of flavonoids and anthocyanins **B**. the terpenoid pathway that leads to the production of terpenes and, via the cleavage of carotenes, C13-norisoporenoids and **C**. the lipoxygenase pathway that leads to the production of volatile aldehydes, alcohols and thiols. Genes selected for targeted expression analysis are indicated in red.
