# Supplementary material for: The Effect of Water Deficit on Two Greek Vitis vinifera L. Cultivars: Physiology, Grape Composition and Gene Expression during Berry Development
Source: Plants (Basel). 2021 Sep 18;10(9):1947. doi: 10.3390/plants10091947 (PMC8470430; doi:10.3390/plants10091947)
Supplement: Supplementary file 1 [file plants-10-01947-s001.zip › plants-1376485-supplementary/Table S1.pdf]

Suppl. Table S1. List of primers used in RT-qPCR analysis

Gene Name	Gene ID	NCBI Accession Number	Forward primer	Reverse primer
VviPAL	VIT_13s0019g04460	XM_010660093.2	GTGAGGGAAGAACTGGGAGC	TTGTCACACTCTTCACCGGG
VviC4H	VIT_06s0004g08150	XM_002266202.3	GAACCACCTGAACCTCTCCG	ATCCGAACTCCACTCCCTGA
VviUFGT	VIT_04s0023g01290	JF522529	TGGTGGCTGACGCATTCAT	CCCCATCTCTGCTGCCATATC
VviDXS	VIT_05s0020g02130	XM_002277883.4	CCAAGGGCGTTACCAAACAG	TCAACTTTTGCAGCCAATTCA
VviCCD1	VIT_13s0064g00840	NM_001280915	TGGACAGCATAGCCAAGGTG	GCTCACGAGGGACAAAGACA
VviLOXA	VIT_06s0004g01450	NM_001281094.1	GCAAATCAAAGGGACAACGCTGTATGG	TGCTTCCACTGCGGCTTCC
VviGGT	VIT_11s0016g02830	XM_002280154.3	TGGCAACAGCTTAGAGGCAGTA	CCCACCTGCCTTTCTCACAT
VviEF1a	VIT_06s0004g03240	XM_002284888.3	GAACTGGGTGCTTGATAGGC	AACCAAAATATCCGGAGTAAAAGA
